# Supplementary material for: Proteomic screens of SEL1L-HRD1 ER-associated degradation substrates reveal its role in glycosylphosphatidylinositol-anchored protein biogenesis
Source: Nat Commun. 2024 Jan 22;15:659. doi: 10.1038/s41467-024-44948-2 (PMC10803770; doi:10.1038/s41467-024-44948-2)
Supplement: Supplementary file 1 — Supplementary Information [file 41467_2024_44948_MOESM1_ESM.pdf]

Supplementary Information

Proteomic screens of SEL1L-HRD1 ER-associated degradation substrates reveal its role in glycosylphosphatidylinositol-anchored protein biogenesis

Xiaoqiong Wei[1,2 #], You Lu[2,3 #], Liangguang Leo Lin[1,2 #], Chengxin Zhang[4], Xinxin Chen[1,2], Siwen Wang[2], Shuangcheng Alivia Wu[1,2], Zexin Jason Li[1,5], Yujun Quan[1], Shengyi Sun[6], Ling Qi[1,2*]

Correspondence: xvr2hm@virginia.edu

This file includes:

Figures S1 to S8

Supplementary figures

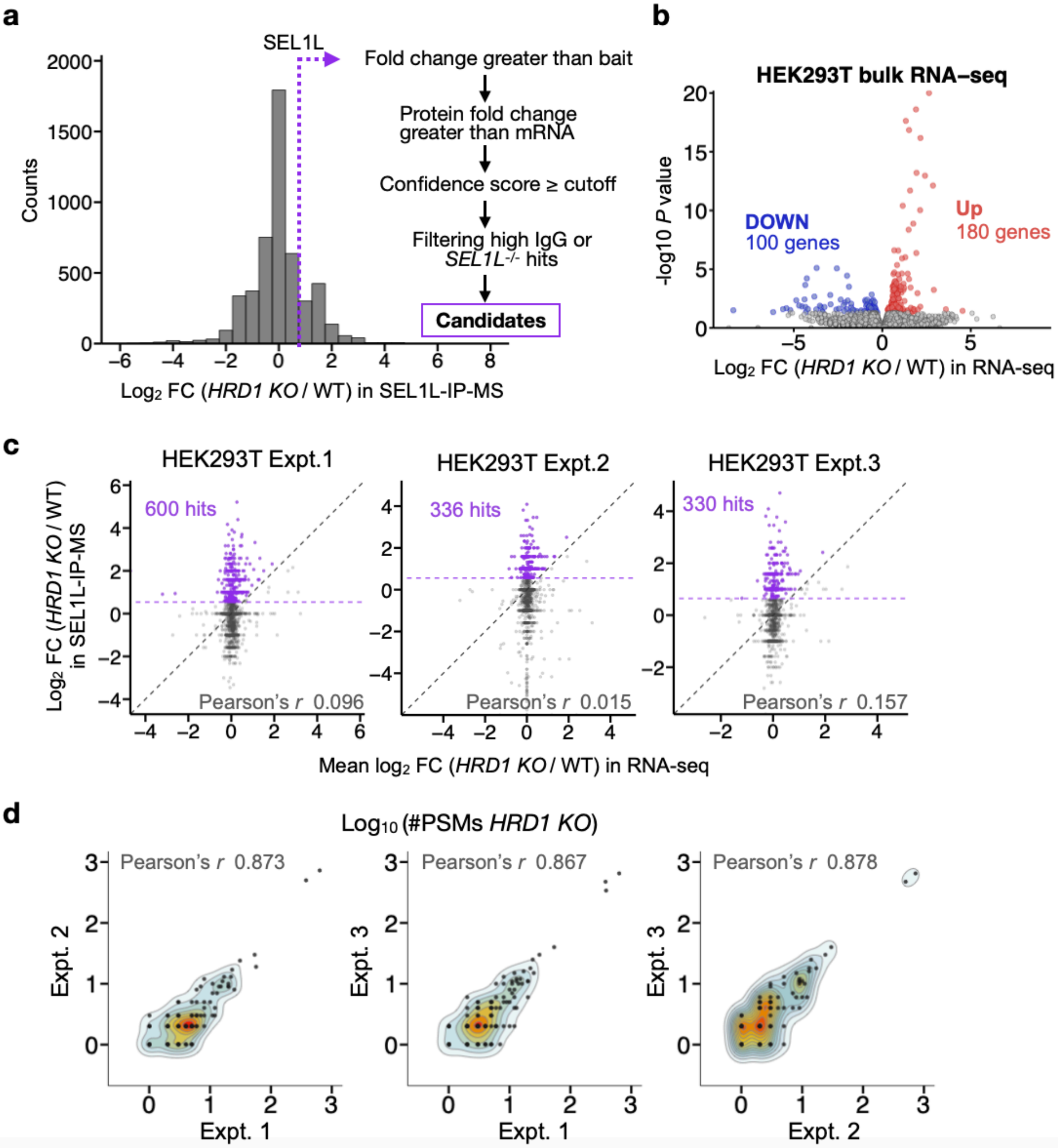

Supplementary Figure 1. Proteomic screening in HEK293T cells.

a, Histogram of the ratio of PSMs in *HRD1 KO* to WT samples for all proteins from the SEL1L-IP-MS data in HEK293T cells from three independent experiments. The ratio calculated from the bait (SEL1L) and indicated by the purple dotted line was used as a cutoff for selecting putative ERAD substrates. Hits that passed this threshold were subsequently filtered based on mRNA fold change, confidence score and PSMs in negative control samples (see Methods for more

details). **b,** Volcano plot showing up- and down-regulated genes in red and blue, respectively, detected in HEK293T using RNA-Seq and the standard DESeq2 pipeline with *P*-values < 0.05 (n=3). **c,** Scatter plot of *HRD1 KO* to WT fold change (FC) of each SEL1L-IP-MS hit and its corresponding mean transcript abundance changes in RNA-seq experiments (n=3). The purple horizontal line represents the bait FC in each SEL1L-IP-MS experiment. Hits above the grey diagonal line exhibit a greater protein abundance change than transcript abundance change. Pearson correlation coefficients (*r*) are shown in each plot. **d,** Correlation of the PSM values of the identified substrate candidates in the *HRD1 KO* samples between experiments. Pearson correlation coefficients (*r*) are indicated in each plot. Contour lines represent density, where red indicates more data points within per unit area.

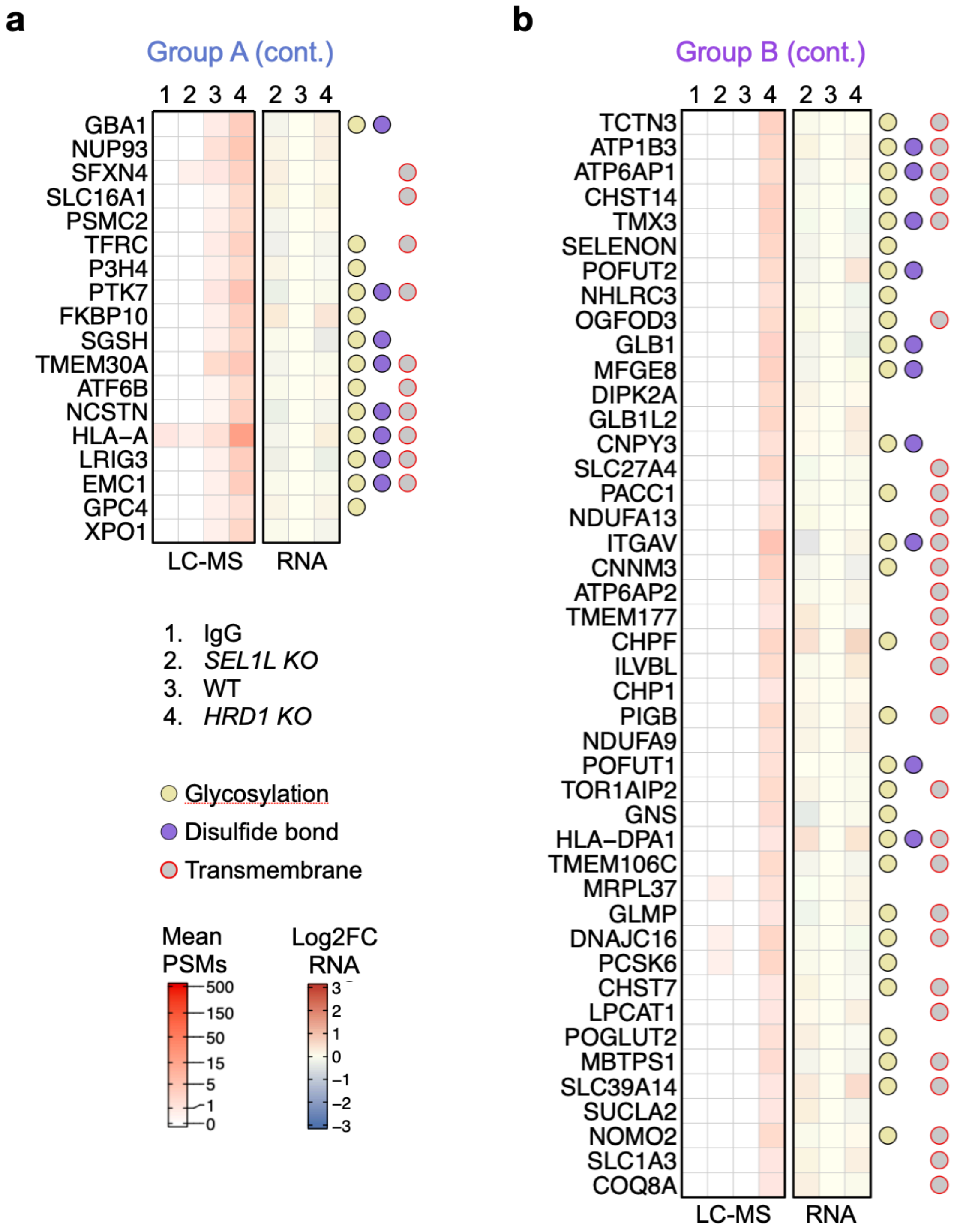

Supplementary Figure 2. SEL1L-HRD1 ERAD substrate candidates in HEK293T cells. **a-b,** Heatmaps showing the mean PSMs from SEL1L-IP-MS samples and RNA log2 fold change (FC) for the remaining ERAD substrates in Group A (**a**) and Group B (**b**) as shown in **Fig. 2d** and e, respectively. Dot plots on the right indicate the presence of protein N-glycosylation, disulfide bonds, and transmembrane domains.

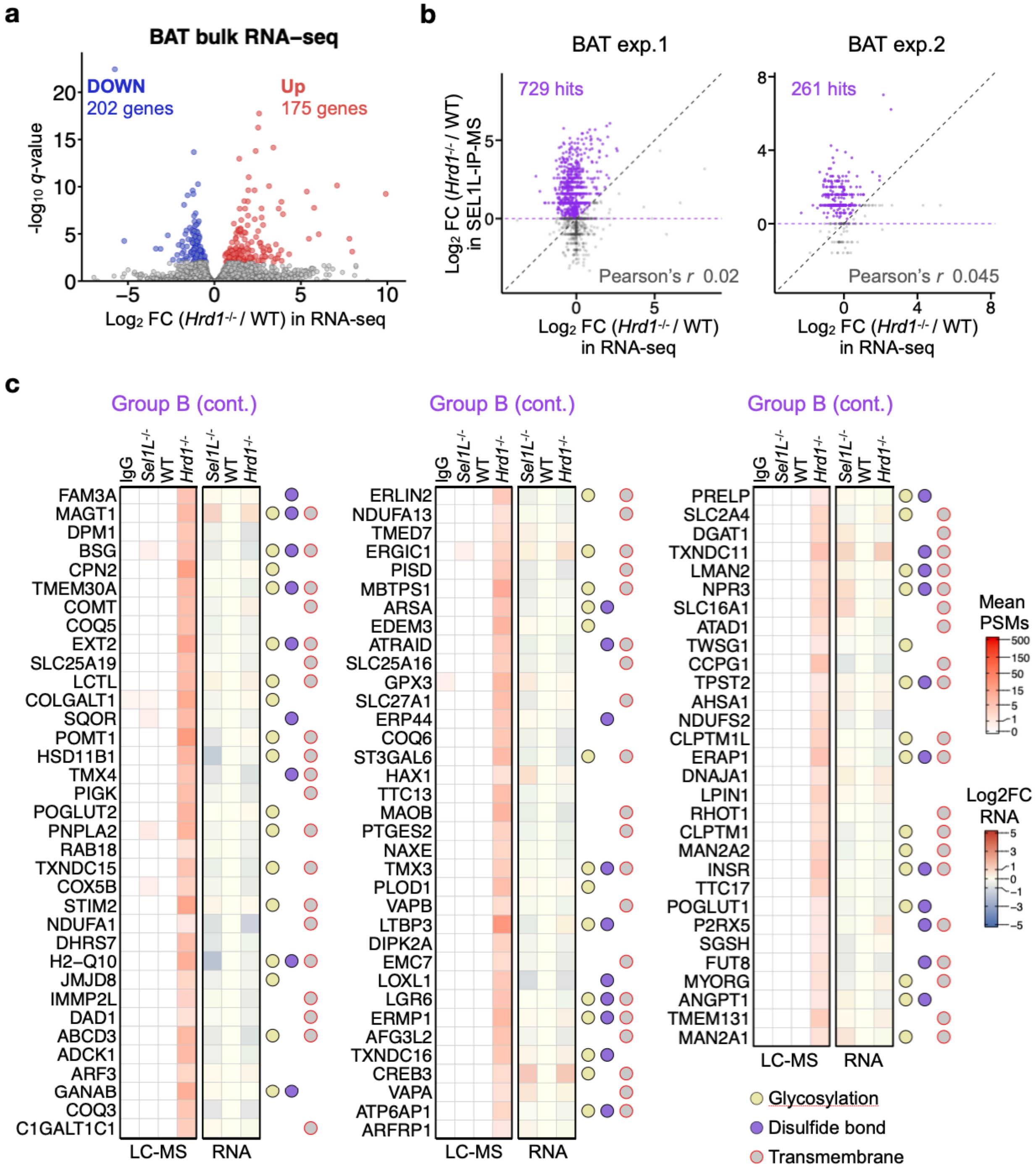

Supplementary Figure 3. Proteomic screening in BAT. a, Volcano plot showing up- and down-regulated genes in red and blue, respectively, detected in BAT using RNA-Seq and the standard DESeq2 pipeline with *q*-values < 0.01 (n=3 for WT and n=2 for *Hrd1*$^{-/-}$). **b,** Scatter plot of *Hrd1*$^{-/-}$ to WT fold change (FC) of each SEL1L-IP-MS hit and its corresponding mean transcript abundance changes in RNA-seq experiments (n=3). The purple horizontal line

represents the bait FC in each SEL1L-IP-MS experiment. Hits above the grey diagonal line have a protein abundance change greater than the transcript abundance change. Pearson correlation coefficients (*r*) are shown in each plot. **c,** Heatmaps presenting the mean PSMs from SEL1L-IP-MS samples and RNA log2 fold change (FC) for the remaining ERAD substrate candidates in Group B shown in Fig. **3d**. Dot plots on the right indicate the presence of protein N-glycosylation, disulfide bonds, and transmembrane domains.

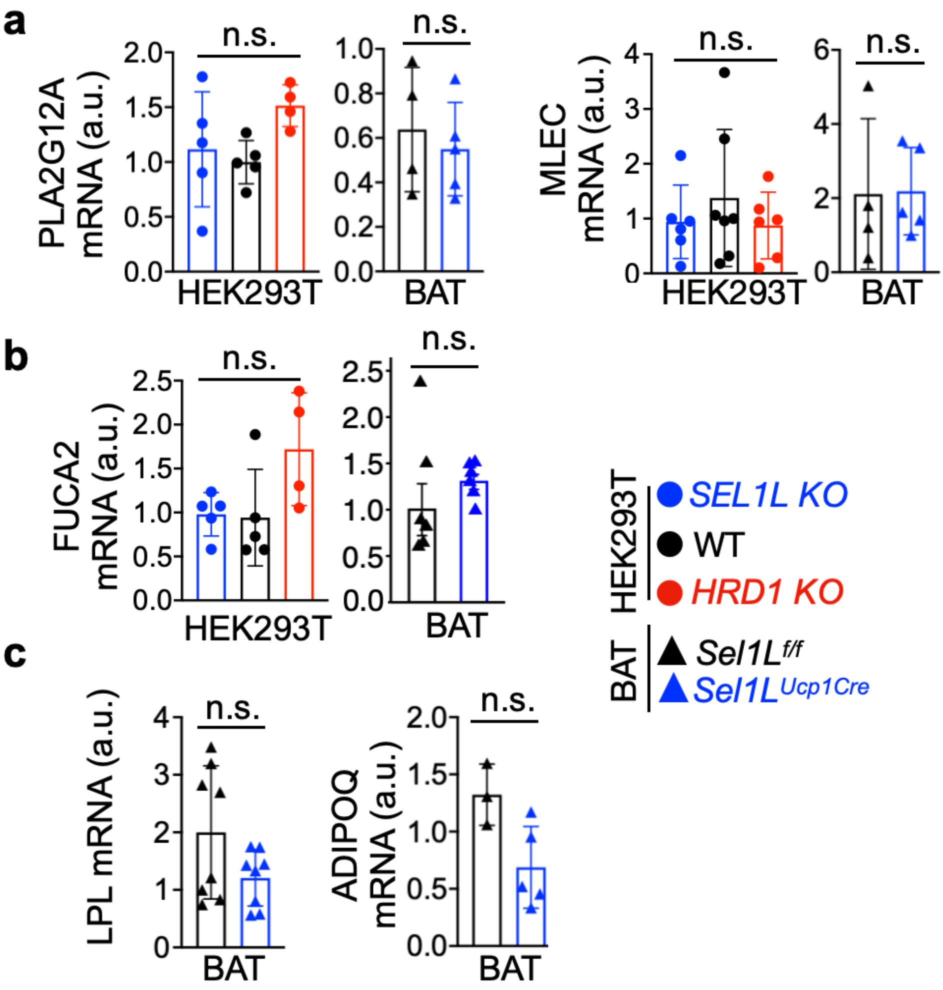

Supplementary Figure 4. mRNA levels of ERAD substrate candidates.

a-b, Relative transcript abundance of common ERAD substrates PLA2G12A and MLEC (a) and FUCA2 (b) in HEK293T cells and BAT (in HEK293T cells, for PLA2G12A, n=5 independent samples for *SEL1L KO* and WT, 4 for *HRD1 KO*; for MLEC, n=7 for WT, 6 for *SEL1L KO* and *HRD1 KO*; for FUCA2, n=5 for WT and *SEL1L KO*, 4 for *HRD1 KO*; for BAT, n=4 mice for WT and 5 for $Sel1L^{Ucp1Cre}$). **c,** Relative transcript abundance of LPL and ADIPOQ in BAT (for LPL, n=8 mice; for ADIPOQ, n=3 mice for WT and 5 for $Sel1L^{Ucp1Cre}$). a.u., arbitrary units. Values represent mean ± SEM. n.s., not significant using one-way ANOVA (for HEK293T) and Student's *t* test (for BAT).

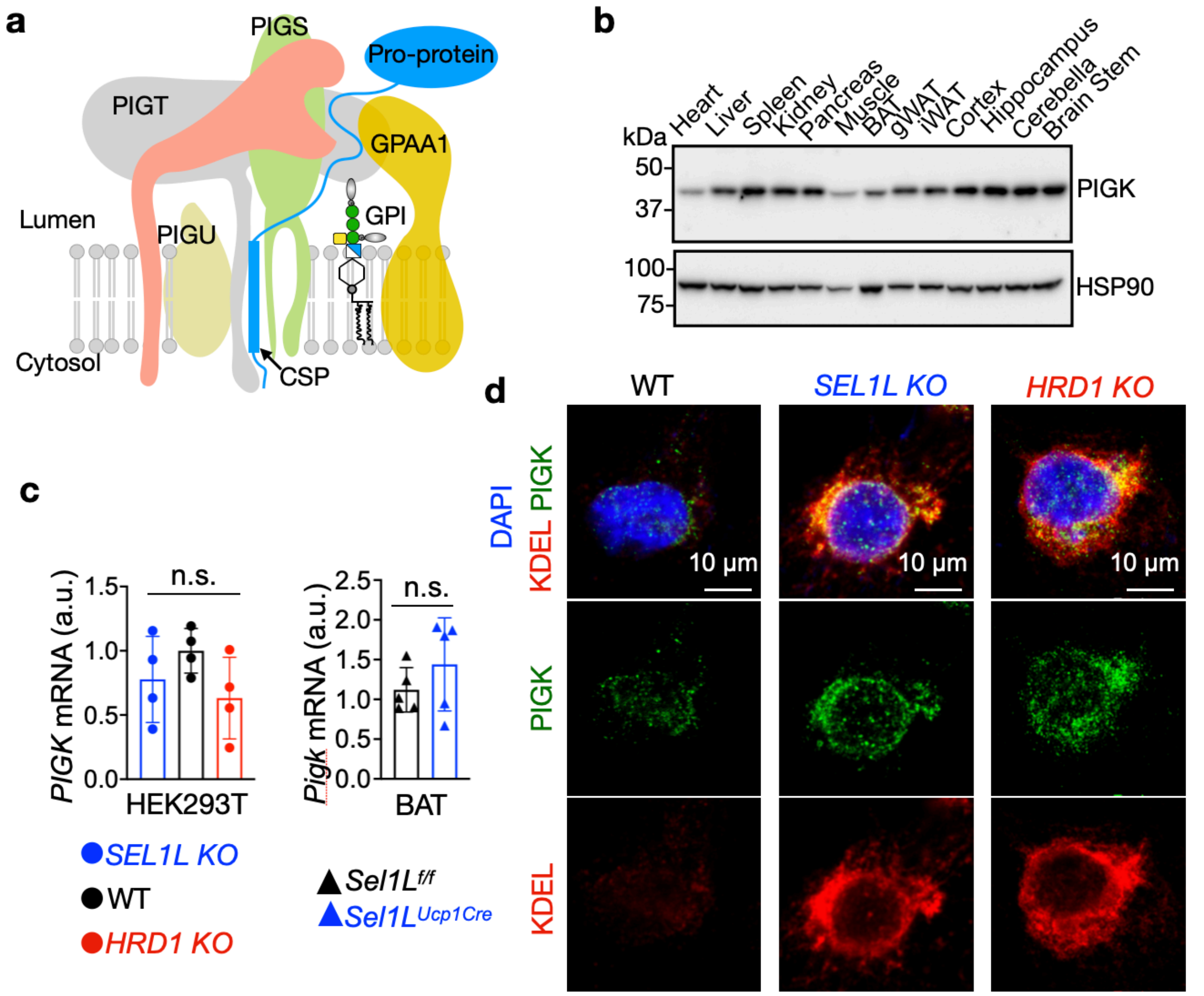

Supplementary Figure 5. PIGK is an endogenous substrate of ERAD.

a, Schematic diagram of the GPI-transamidase complex. PIGK catalyzes the transfer of mature GPI to the carboxyl terminus of precursor proteins after cleavage of the C-terminal signal peptide (CSP) (PIGK, pink; PIGT, gray; PIGS, green; PIGU, light yellow; GPAA1, yellow; pro-protein, blue). **b,** Immunoblotting of endogenous PIGK in various tissues from WT C57BL/6J mice. **c,** Quantitative PCR analyses of *PIGK* mRNA levels in HEK293T cells (n=4 from 2 independent repeats), and BAT (n=5 from 2 independent repeats). a.u., arbitrary units. Values represent mean ± SEM. n.s., not significant by one-way ANOVA (for HEK293T) and Student's *t* test (for BAT). **d,** Representative confocal images of PIGK (green), DAPI (blue) and KDEL (red) in HEK293T cells. Of note, both KDEL and PIGK were elevated in the absence of ERAD. Images were acquired under the same microscope parameters.

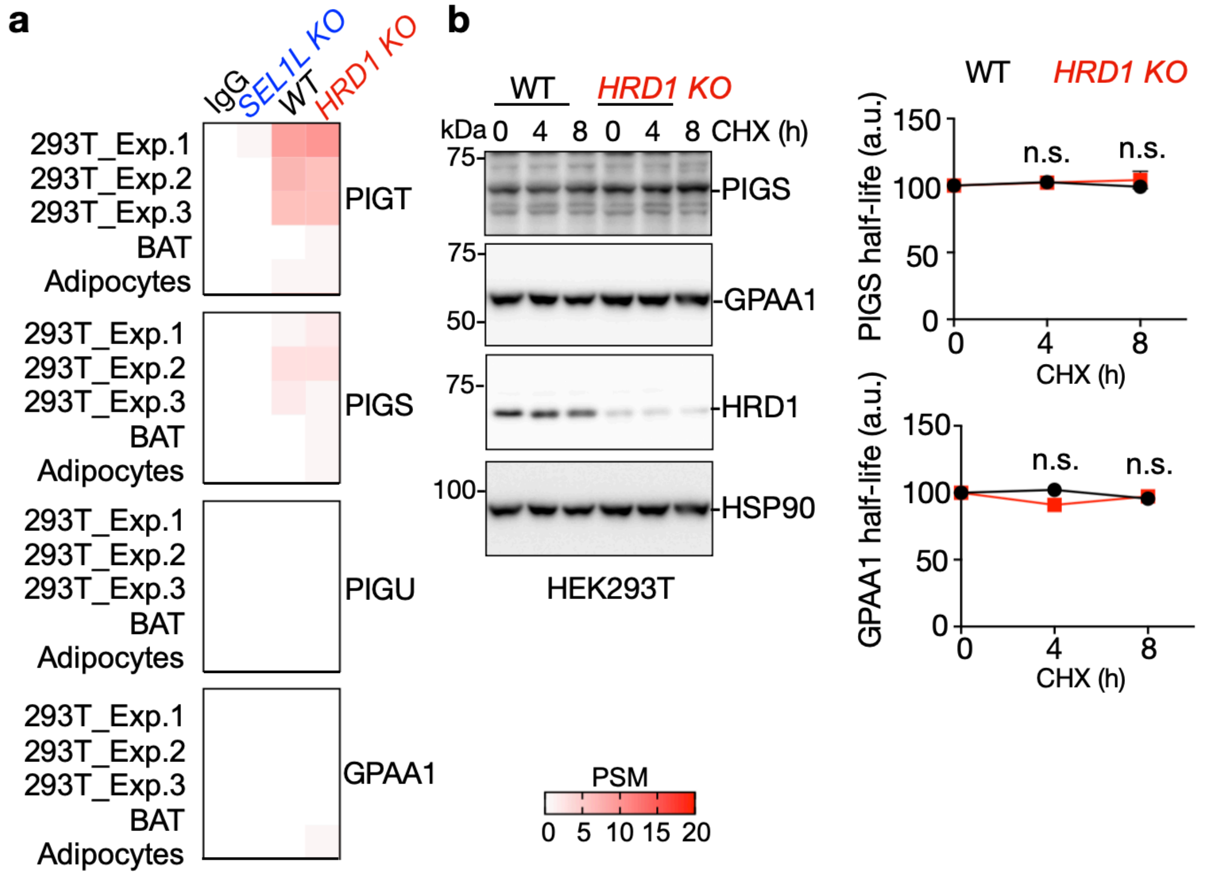

Supplementary Figure 6. SEL1L-HRD1 ERAD specifically targets PIGK, not the other subunits of the GPI-transamidase complex.

a, Heatmaps showing the PSMs of PIGT, PIGS, PIGU and GPAA1 from SEL1L-IP-MS experiments in HEK293T cells, BAT and differentiated brown adipocytes. **b,** Representative immunoblot analyses of endogenous GPI-transamidase components in HEK293T cells treated with 50 μg/ml cycloheximide (CHX) for the indicated times with quantitation shown on the right (n=3 independent repeats for PIGS, 2 for GPAA1). a.u., arbitrary units. Values represent mean ± SEM. n.s., not significant using Student's t test.

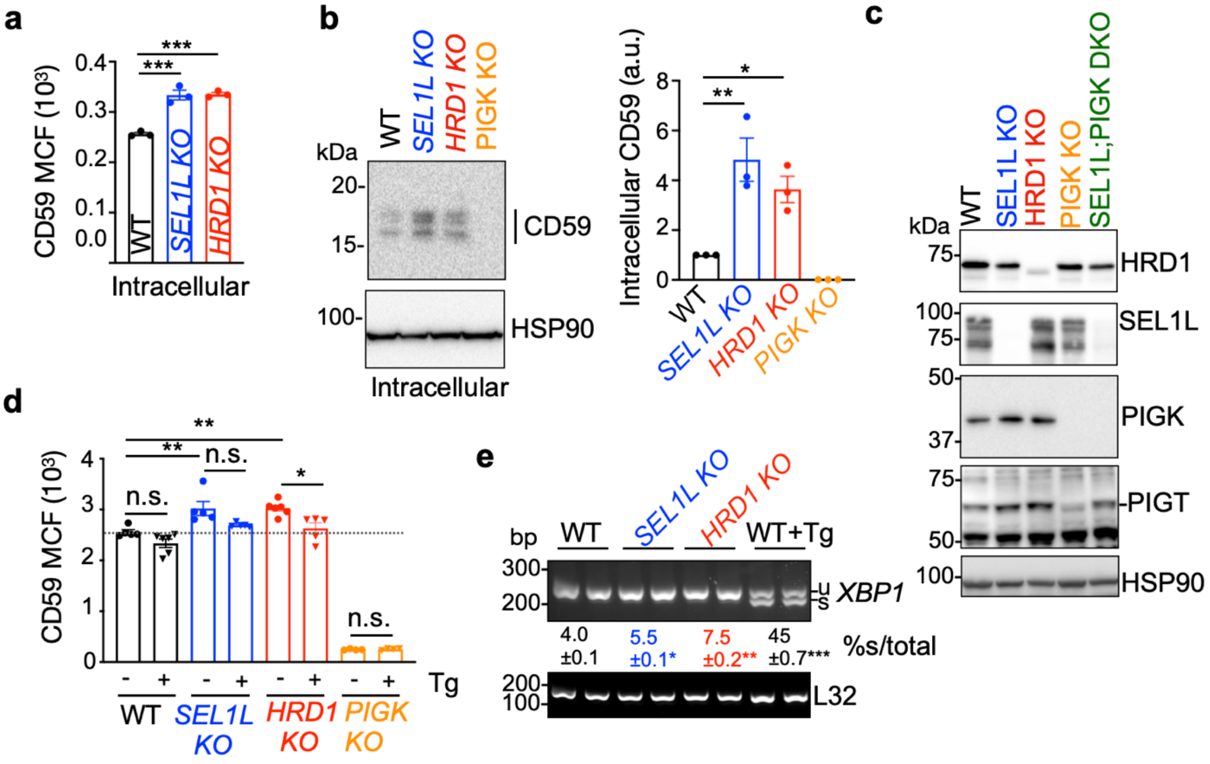

Supplementary Figure 7. Generation of various cell lines and the effect of ER stress on surface CD59 levels. a, Quantitation of the flow cytometric analysis of intracellular CD59 in HEK293T cells treated with 5 U/mL phosphatidylinositol-specific phospholipase C (PI-PLC) to deplete surface GPI-anchored proteins (n=3 independent repeats). **b**, Representative immunoblotting of intracellular CD59 in HEK293T cells treated with PI-PLC (n=3 independent repeats). a.u., arbitrary units. **c**, Immunoblotting validation of various KO HEK293T cells (n=2 independent repeats). **d,** Quantitation for flow cytometric analysis of surface CD59 in HEK293T cells treated with or without 50 nM thapsigargin (Tg) for 4 hours (n=3 independent repeats). **e.** RT-PCR of *XBP1* splicing. u/s, unspliced/spliced *Xbp1*. Quantitation of the relative abundance of spliced *Xbp1* bands was indicated below (n=2 independent repeats). Values represent mean ± SEM. n.s., not significant * $P < 0.05$, ** $P < 0.01$, *** $P < 0.001$ using one-way ANOVA followed by Dunnett's multiple comparisons test.

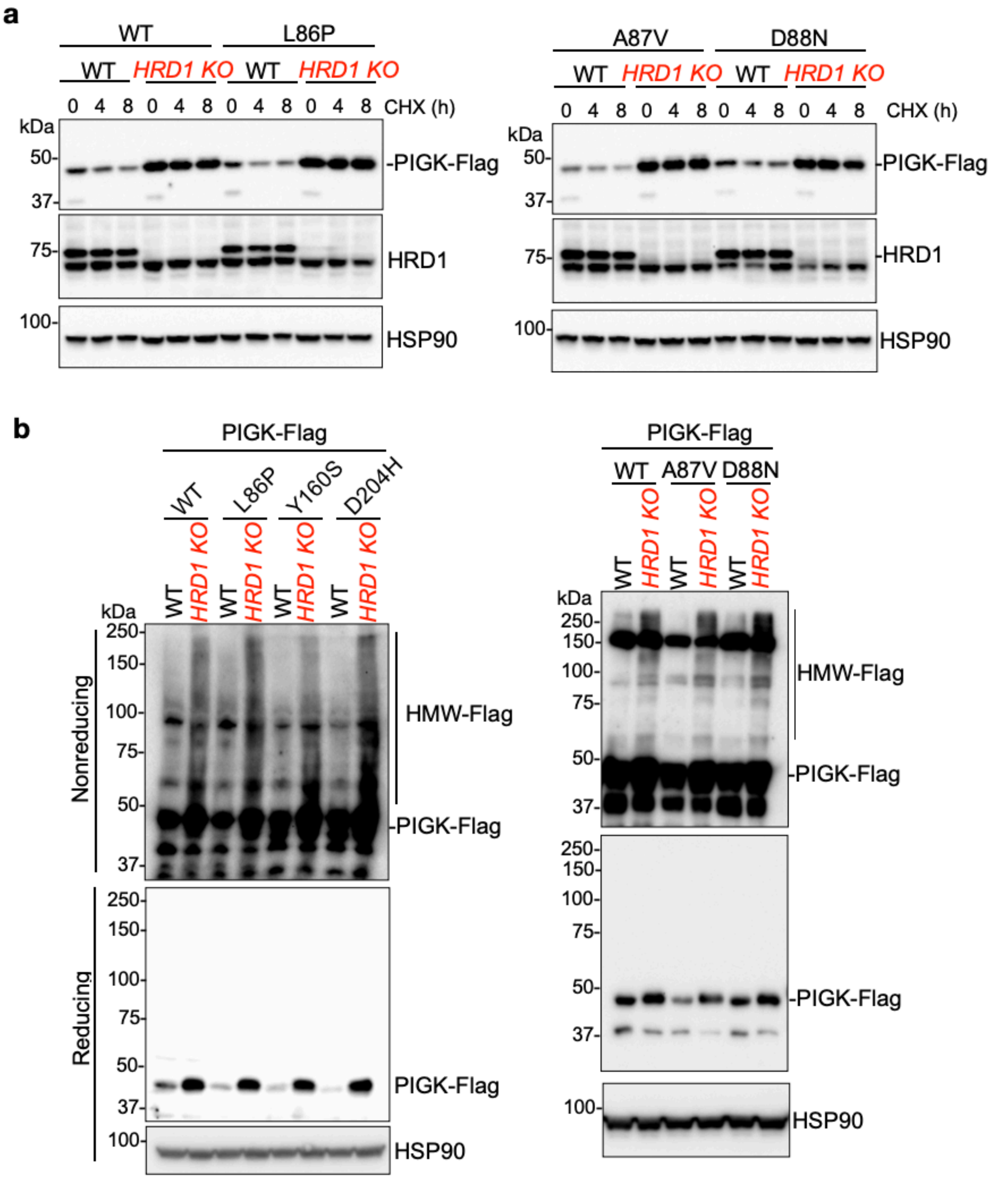

Supplementary Figure 8. PIGK disease mutants are SEL1L-HRD1 ERAD substrates.

a, Immunoblot analyses of WT and mutant PIGK in transfected WT and *HRD1 KO* HEK293T cells treated with CHX for 0, 4 and 8 hours with quantitation shown in Fig. 8d. **b,** Immunoblot analyses of high molecular weight (HMW) aggregates of WT and mutant PIGK in transfected WT and *HRD1 KO* HEK293T cells under nonreducing and reducing conditions (n=3 independent repeats).
