# Supplementary material for: Proteomic screens of SEL1L-HRD1 ER-associated degradation substrates reveal its role in glycosylphosphatidylinositol-anchored protein biogenesis
Source: Nat Commun. 2024 Jan 22;15:659. doi: 10.1038/s41467-024-44948-2 (PMC10803770; doi:10.1038/s41467-024-44948-2)
Supplement: Supplementary file 3 — Reporting Summary [file 41467_2024_44948_MOESM3_ESM.pdf]

nature portfolio

Corresponding author(s): Ling Qi

Last updated by author(s): Dec 17, 2023

Reporting Summary

Nature Portfolio wishes to improve the reproducibility of the work that we publish. This form provides structure for consistency and transparency in reporting. For further information on Nature Portfolio policies, see our Editorial Policies and the Editorial Policy Checklist.

Statistics

For all statistical analyses, confirm that the following items are present in the figure legend, table legend, main text, or Methods section.

n/a	Confirmed	
☐	☒	The exact sample size (*n*) for each experimental group/condition, given as a discrete number and unit of measurement
☐	☒	A statement on whether measurements were taken from distinct samples or whether the same sample was measured repeatedly
☐	☒	The statistical test(s) used AND whether they are one- or two-sided<br>*Only common tests should be described solely by name; describe more complex techniques in the Methods section.*
☒	☐	A description of all covariates tested
☐	☒	A description of any assumptions or corrections, such as tests of normality and adjustment for multiple comparisons
☐	☒	A full description of the statistical parameters including central tendency (e.g. means) or other basic estimates (e.g. regression coefficient) AND variation (e.g. standard deviation) or associated estimates of uncertainty (e.g. confidence intervals)
☐	☒	For null hypothesis testing, the test statistic (e.g. *F*, *t*, *r*) with confidence intervals, effect sizes, degrees of freedom and *P* value noted<br>*Give P values as exact values whenever suitable.*
☒	☐	For Bayesian analysis, information on the choice of priors and Markov chain Monte Carlo settings
☒	☐	For hierarchical and complex designs, identification of the appropriate level for tests and full reporting of outcomes
☐	☒	Estimates of effect sizes (e.g. Cohen's *d*, Pearson's *r*), indicating how they were calculated

Our web collection on statistics for biologists contains articles on many of the points above.

Software and code

Policy information about availability of computer code

Data collection	Quantitative PCR was performed by CFX Opus 384 Real-Time PCR System (Bio-Rad, 12011452).<br>All the RNA-seq libraries were sequenced pair-ended for 151 cycles on the same flow cell using a NovaSeq 6000 instrument (Illumina).<br>Flow cytometry was performed with the Attune Flow Cytometer.<br>Immunofluorescence images were collected by Leica STELLARIS 8 FALCON Confocal Microscope<br>PIGK structure was predicted using AlphaFold2 (https://alphafold.ebi.ac.uk/).<br>Mass spectrum data were collected with Q exactive HF mass spectrometer (Thermo Scientific, San Jose CA) using an EasySpray source.
Data analysis	The target sites for CRISPR/Cas9 were selected using the web program (http://www.e-crisp.org/E-CRISP/designcrispr.html);<br>Statistics tests were performed in GraphPad Prism version 9.0 (GraphPad Software);<br>Immunofluorescence images were analyzed using the Fiji 2.0.0 software (Image J)<br>Proteins were identified by searching the MS/MS data against UniProt entries using Proteome Discoverer (v2.4, Thermo Scientific). Search parameters included MS1 mass tolerance of 10 ppm and fragment tolerance of 0.2 Da;

For manuscripts utilizing custom algorithms or software that are central to the research but not yet described in published literature, software must be made available to editors and reviewers. We strongly encourage code deposition in a community repository (e.g. GitHub). See the Nature Portfolio guidelines for submitting code & software for further information.

Data

Policy information about availability of data

All manuscripts must include a data availability statement. This statement should provide the following information, where applicable:

- Accession codes, unique identifiers, or web links for publicly available datasets
- A description of any restrictions on data availability
- For clinical datasets or third party data, please ensure that the statement adheres to our policy

The IP-MS datasets for mouse brown adipose tissues and HEK293T cells are available via ProteomeXchange with identifiers PXD041803 and PXD041882, respectively. The high-throughput sequencing data have been deposited to the Gene Expression Omnibus (GEO) database under the accession number GSE231583. The materials and reagents used are either commercially available or available upon request. All other data are available in the main text or in the supplementary information and Source Data Files. Source data are provided with the paper.

Research involving human participants, their data, or biological material

Policy information about studies with human participants or human data. See also policy information about sex, gender (identity/presentation), and sexual orientation and race, ethnicity and racism.

Reporting on sex and gender	N/A
Reporting on race, ethnicity, or other socially relevant groupings	N/A
Population characteristics	N/A
Recruitment	N/A
Ethics oversight	N/A

Note that full information on the approval of the study protocol must also be provided in the manuscript.

Field-specific reporting

Please select the one below that is the best fit for your research. If you are not sure, read the appropriate sections before making your selection.

☒ Life sciences ☐ Behavioural & social sciences ☐ Ecological, evolutionary & environmental sciences

For a reference copy of the document with all sections, see nature.com/documents/nr-reporting-summary-flat.pdf

Life sciences study design

All studies must disclose on these points even when the disclosure is negative.

Sample size	Sample size was determined based on the formula of the power analysis, N=8(CV)^2[1+(1-PC)^2]/(PC)^2, to reach the error = 0.05, Power = 0.80, percentage change in means (PC) = 20%, co-efficient of variation (CV) = 10 ~ 15% (varies between the experiments). The sample size for each animal experiment was described in the figure legend.
Data exclusions	No animals or samples were excluded from the analysis.
Replication	All experiments were repeated at least twice or performed with independent samples. The details were described in the figure legend, methods part and section "Statistical Analysis". Source data was provided with the paper.
Randomization	Mice were randomly assigned based on the age, genotype and gender. Cells were grown under the same conditions and randomly allocated into different groups without any bias.
Blinding	When experiments were done by one investigator, blindness is not applied or possible. When experiments are done sequentially by different investigators, investigators were blinded to allocation during experiments and outcome assessment.

Reporting for specific materials, systems and methods

We require information from authors about some types of materials, experimental systems and methods used in many studies. Here, indicate whether each material, system or method listed is relevant to your study. If you are not sure if a list item applies to your research, read the appropriate section before selecting a response.

Materials & experimental systems

n/a	Involved in the study	
☐	☒	Antibodies
☐	☒	Eukaryotic cell lines
☒	☐	Palaeontology and archaeology
☐	☒	Animals and other organisms
☒	☐	Clinical data
☒	☐	Dual use research of concern
☒	☐	Plants

Methods

n/a	Involved in the study	
☒	☐	ChIP-seq
☐	☒	Flow cytometry
☒	☐	MRI-based neuroimaging

Antibodies

Antibodies used

Western Blot antibodies: anti-HSP90 (Santa Cruz, #sc-13119, 1:5,000), anti-SEL1L (home-made, 1:10,000), anti-HRD1 (Proteintech, #13473-1, 1:2,000), anti-OS9 (Abcam, #ab109510, 1:5,000), anti-PLA2G12A (Proteintech,16009-1-AP, 1:2000), anti-MLEC (Proteintech, 26655-1-AP, 1:1000), anti-FUCA2 (Proteintech, 15157-1-AP, 1:1000), anti-LPL (Novus Biologicals, AF7197, 1:500), anti-ADIPOQ (Proteintech 21613-1-AP), anti-PIGK (Abcam, ab201693, 1:2000), anti-Ubiquitin (Santa Cruz, sc-8017, 1:1000), anti-HA (Sigma-Aldrich, H3663, 1:1000), anti-FLAG (Sigma-Aldrich, F1804, 1:1000), anti-PIGT (Proteintech, 16906-1-AP 1:1000), anti-PIGU (Abclonal, A18538, 1:1000), anti-PIGS (Proteintech, 18334-1-AP, 1:1000), anti-GPAA1 (Proteintech, 10104-1-AP, 1:1000).
Flow cytometric antibodies: T5 mAb (BEI Resources, NR-50267, 1:100), FITC anti-human CD59 Antibody (BioLegend, 304706), FLAER Alexa 488 proaerolysin variant (Cedarlane Labs, FL1S-R), PE anti-human CD87 Antibody ((BioLegend, 336906).
Antibodies used for immunoprecipitation were: anti-PIGK (Abcam, ab201693, 2ug/sample), anti-SEL1L (home-made, 2ug/sample), anti-HA (Sigma-Aldrich, H3663, 2ug/sample), anti-Flag (Sigma-Aldrich, F1804, 2ug/sample)
Antibodies used for immunofluorescence were: Anti-PIGK (Abcam, ab201693, 1:200), anti-KDEL (Novus Biologicals, NBP1-97469, 1:500)

Validation

Home made antibody: anti-SEL1L : validated for Western blot (Zhou et al., Science 2020) in mouse and human. We validated the application in immunoprecipitation in HEK293T cells in this study (Figure 1).
Commercial antibodies were validated by the manufactures.
Anti-HSP90 (Santa Cruz, sc-7947): validated for western blot in human and mouse (https://www.scbt.com/p/hsp-90alpha-beta-antibody-h-114).
Anti-HRD1 (ProteinTech, 13473-1): validated for western blot, immunostaining and immunoprecipitation in mouse and human (https://www.ptglab.com/products/SYVN1-Antibody-13473-1-AP.htm).
Anti-OS9 (Abcam, ab109510): validated for western blot and immunostaining in mouse and human (https://www.abcam.com/os9-Antibody-epr42722-ab109510.html).
Anti-PLA2G12A (Proteintech,16009-1-AP): validated for western blot in mouse and human (https://ptglab.com/products/PLA2G12A-Antibody-16009-1-AP.htm)
Anti-MLEC (Proteintech, 26655-1-AP): validated for western blot in mouse and human (https://www.ptglab.com/products/Malectin-Antibody-26655-1-AP.htm).
Anti-FUCA2 (Proteintech, 15157-1-AP): validated for western blot in mouse and human (https://www.ptglab.com/products/FUCA2-Antibody-15157-1-AP.htm)
Anti-KDEL (Novus NBP1-97469): validated for immunostaining in mouse and human (https://www.novusbio.com/products/kdel-antibody-10c3_nbp1-97469).
Anti-LPL (Novus Biologicals, AF7197): validated for western blot in mouse and human (https://www.novusbio.com/products/lipoprotein-lipase-lpl-antibody_af7197).
Anti-ADIPOQ (Proteintech 21613-1-AP): validated for western blot in mouse and human (https://www.ptglab.com/products/ADIPOQ-Antibody-21613-1-AP.htm).
Anti-PIGK (Abcam, ab201693): validated for western blot, immunoprecipitation and immunofluorescence in mouse and human (https://www.abcam.com/products/primary-antibodies/pigk-antibody-epr17843-ab201693.html). We also validated the application in wester blot and immunoprecipitation in HEK293T cells in this study (Figure 6 and Extended Data Figure 7).
Anti-Ubiquitin (Santa Cruz, sc-8017):validated for western blot in HEK293T cells (https://www.scbt.com/p/ubiquitin-antibody-p4d1?requestFrom=search).
Anti-HA (Sigma-Aldrich, H3663): validated for western blot in HEK293T cells (https://www.sigmaaldrich.com/US/en/product/sigma/h3663?cm_sp=Insite-_-caSrpResults_srpRecs_srpModel_h3663-_-srpRecs3-1&lang=en&region=US).
Anti-FLAG (Sigma-Aldrich, F1804): validated for western blot (https://www.sigmaaldrich.com/US/en/product/sigma/f1804?cm_sp=Insite-_-caSrpResults_srpRecs_srpModel_f1804-_-srpRecs3-1&lang=en&region=US).
Anti-PIGT (Proteintech, 16906-1-AP): validated for western blot (https://www.ptglab.com/products/PIGT-Antibody-16906-1-AP.htm)
Anti-PIGU (Abclonal, A18538): validated for western blot (https://abclonal.com/catalog-antibodies/PIGURabbitpAb/A18538).
Anti-PIGS (Proteintech, 18334-1-AP): validated for western blot (https://www.ptglab.com/products/PIGS-Antibody-18334-1-AP.htm)
Anti-GPAA1 (Proteintech, 10104-1-AP): validated for western blot (https://www.ptglab.com/products/GPAA1-Antibody-10104-1-AP.htm).

Eukaryotic cell lines

Policy information about cell lines and Sex and Gender in Research

Cell line source(s)

HEK293T cells were originally obtained from ATCC. Pre-adipocytes were isolated from mouse brown adipose tissue and immortalized using SV40 large T antigen and differentiated into adipocytes, which was indicated in the Method section of the manuscript and reported previously (Zhou et al. Science 2022, and Klein et al., Bioessays 2002).

Authentication	The differentiated adipocytes were authenticated using visual examination of cell morphology, and accumulation of lipid droplets. HEK293T cells were purchased from ATCC (STR profile validated). Cells were maintained at lowest passage numbers.
Mycoplasma contamination	All cell lines used for experiments had no mycoplasma contamination after testing.
Commonly misidentified lines (See ICLAC register)	The cell lines are not listed in the database.

Animals and other research organisms

Policy information about studies involving animals; ARRIVE guidelines recommended for reporting animal research, and Sex and Gender in Research

Laboratory animals	Species: Mus musculus;<br>Sex: Female/Male; Age: 12-13 weeks;<br>Information of sex and age was indicated in the figure legends.<br>Strains:<br>1. All mice were in C57BL/6J background (Sha et al., Cell Metabolism 2014).<br>2. Sel1L flox/flox, Ucp1-Cre mice: Zhou et al., Science 2020.<br>3. Sel1LERCre mice: Zhou et al., Science 2020 and Sun et al., PNAS 2014.<br>The details were also described in the Method section of the manuscript.<br>All mice were housed in a pathogen-free animal facility at 22 ± 1°C on a 12-hr light/dark cycle with 40-60% humidity and fed a low-fat diet (13% fat, 57% carbohydrate, and 30% protein, LabDiet 5LOD), unless otherwise indicated.
Wild animals	This study did not include wild animals.
Reporting on sex	Both males and females were used.
Field-collected samples	This study did not include field-collected samples.
Ethics oversight	All animal procedures were approved by and done in accordance with the the Institutional Animal Care & Use Committee (IACUC) at the University of Michigan Medical School (PRO00010658).

Note that full information on the approval of the study protocol must also be provided in the manuscript.

Flow Cytometry

Plots

Confirm that:

☒ The axis labels state the marker and fluorochrome used (e.g. CD4-FITC).

☒ The axis scales are clearly visible. Include numbers along axes only for bottom left plot of group (a 'group' is an analysis of identical markers).

☒ All plots are contour plots with outliers or pseudocolor plots.

☒ A numerical value for number of cells or percentage (with statistics) is provided.

Methodology

Sample preparation	The HEK293T cells were harvested and incubated with anti-human CD59 antibody conjugated with FITC (BioLegend 304706, 1:100) or FLAER (Alexa 488 proaerolysin variant, 1:100) on ice for 25 minutes. After two washes with PBS, the cells were analyzed with an Attune Flow Cytometer (ThermoFisher Scientific). For free GPI test, the cells were incubated with T5 mAb (BEI Resources, NR-50267, 1:100) on ice for 25 minutes. The cells were then washed twice followed by staining with Alexa Fluor 647-conjugated goat anti-mouse IgM secondary antibody (Invitrogen A-21238, 1:1000) and analyzed with the Attune Flow Cytometer.
Instrument	Attune Flow Cytometer (ThermoFisher Scientific).
Software	Attune Flow Cytometer
Cell population abundance	At least 10,000 cells were collected for FACS analysis.
Gating strategy	Gates were clearly described in the method.

☒ Tick this box to confirm that a figure exemplifying the gating strategy is provided in the Supplementary Information.
