# Supplementary material for: Proteomic screens of SEL1L-HRD1 ER-associated degradation substrates reveal its role in glycosylphosphatidylinositol-anchored protein biogenesis
Source: Nat Commun. 2024 Jan 22;15:659. doi: 10.1038/s41467-024-44948-2 (PMC10803770; doi:10.1038/s41467-024-44948-2)
Supplement: Supplementary file 4 — Description of Additional Supplementary Files [file 41467_2024_44948_MOESM4_ESM.pdf]

Description of Additional Supplementary Files

File name: Supplementary Data 1
Description: High-confidence endogenous SEL1L-HRD1 ERAD substrates in HEK293T cells. Total 119 positive hits were identified in at least two independent repeats.

File name: Supplementary Data 2
Description: High-confidence endogenous SEL1L-HRD1 ERAD substrates in brown adipose tissue. Total 152 positive hits were identified in at least two independent repeats.

File name: Supplementary Data 3
Description: Comparative analyses between HEK293T cells and brown adipose tissue. Total 238 shared or cell-type-specific ERAD substrates and their corresponding pathway analysis.
